# Exploration of the content validity and feasibility of the EQ-5D-3L, ICECAP-O and ASCOT in older adults

**DOI:** 10.1186/s12913-015-0862-8

**Published:** 2015-05-15

**Authors:** Karen M van Leeuwen, Aaltje P D Jansen, Maaike E Muntinga, Judith E Bosmans, Marjan J Westerman, Maurits W van Tulder, Henriette E van der Horst

**Affiliations:** Department of General Practice and Elderly Care Medicine and EMGO Institute for Health and Care Research, VU University Medical Center, Van der Boechorststraat 7, 1081 BT Amsterdam, The Netherlands; Department of Medical Humanities and EMGO Institute for Health and Care Research, VU University Medical Center, Van der Boechorststraat 7, 1081 BT Amsterdam, The Netherlands; Department of Health Sciences and EMGO Institute for Health and Care Research, Faculty of Earth and Life Sciences, VU University, De Boelelaan 1085, 1081 HV Amsterdam, The Netherlands

**Keywords:** Preference-based measures, Quality of life, Content validity, Feasibility, Older adults

## Abstract

**Background:**

In economic evaluations of care services for older adults health-related quality of life (QoL) measures such as the EQ-5D are increasingly replaced by the ICECAP-O and ASCOT, which cover a broader scope of QoL than health alone. Little is known about the content validity and feasibility of these measures. The purpose of this study was to explore the content validity and feasibility of the EQ-5D-3L, ICECAP-O and ASCOT in older adults.

**Methods:**

Ten older adults were purposively sampled using a maximum variation principle. Think-aloud and verbal probing techniques were used to identify response issues encountered during the interpretation of items and the selection of response options. We used constant comparative methods to analyse the data.

**Results:**

Two types of response issues were identified for various items in all three measures: interpretation issues and positive responses. Issues with the mapping of a response on one of the response options were least often encountered for the EQ-5D-3L items. Older adults considered the items of the ICECAP-O and ASCOT valuable though more abstract than the EQ-5D-3L.

**Conclusions:**

Researchers who intend to use the EQ-5D, ICECAP-O or ASCOT in economic evaluations of care services for older adults, should be aware of the response issues that occur during the administration of these measures. Older adults perceived none of the measures as providing a comprehensive picture of their QoL. A preference from older adults for one of the measures depends on the extent to which the items reflect current personal concerns in life.

## Background

Care services for older adults are not primarily aimed at improving health, but rather at compensating health declines to preserve more general quality of life (QoL) aspects such as independence, daily functioning and social participation. Concerns have been expressed that benefits of these care services are underestimated in economic evaluations when health-related QoL measures such as the EQ-5D are used [1–5]. Valuable aspects of care services for older adults may consequently be overlooked in policy making processes informed by these evaluations.

To address these concerns researchers recently developed the ICEpop CAPability measure for Older people (ICECAP-O) [6] and the Adult Social Care Outcomes Toolkit (ASCOT) [7], which include a broader set of QoL aspects. These measures were developed for the purpose of evaluating health and social care services and can be used in economic evaluations. Because of the inclusion of QoL aspects beyond health and the involvement of older adults in the development of the measures [3, 6, 7], it is likely that the ICECAP-O and ASCOT more adequately reflect the objectives of care services for older adults and the perspective of older adults than the EQ-5D [8].

When evaluating care services for older adults, it is important that the measures used validly assess the QoL of older adults and are feasible to use in samples of older populations. The measure should be comprehensive and adequately reflect the patient perspective (i.e. *content validity* [9, 10]), and the items should be comprehensible and acceptable; the target population should be able and willing to give responses to the questions (i.e. *feasibility*). Poor content validity and feasibility increase the risk of misclassification, biased results, missing responses and irritation, boredom or intellectual withdrawal of respondents [11–13].

The coverage of relevant QoL aspects and the operationalization of these aspects determine the content validity and feasibility of QoL measures. Aspects should be operationalized in such a way that older adults understand what is meant and that the items and response options correspond to the reality of their daily life. Previously, researchers that investigated survey response processes have shown that answers to questions are prone to a variety of response issues and that responses not always correspond with the developers’ intentions [12, 14–21]. Response issues are a result of the interplay between the respondents’ motivation and capacities, the interview setting and attributes of the measurement instrument [14, 17, 20, 22] and can arise during several stages of the response process, as presented in a model by Tourangeau [17]. These issues may threat the validity and feasibility of the measures [20, 22, 23]. For example, commonly reported problems concern interpretation difficulties due to double-barrelled or ambiguous questions, and response tendencies such as positive responding and acquiescence. In the case of QoL of older adults, adaptation and comparison mechanisms pose a challenge to measurement and may cause unexpected ways of responding [12, 24–28].

Qualitative methods are increasingly used to include the target populations’ perspective on the content validity and feasibility of measures [9, 12, 18, 21, 23, 29, 30]. Cognitive interviewing techniques such as the think-aloud technique provide insight in the response process. Threats to content validity and feasibility of measures can be revealed when the nature of response issues and the meaning of responses are identified and better understood [9, 18, 21, 22, 29, 31].

Little is known yet about the extent to which response issues emerge using the ICECAP-O or ASCOT, and whether this differs from the EQ-5D. Although the items were in general interpreted as intended, some struggles with the ASCOT and the adult version of the ICECAP (ICECAP-A) were reported [7, 32, 33]. Compared to the EQ-5D, more subjective and diverse interpretations of the terms in the ICECAP-A were found [32]. A few studies showed that the ICECAP-A and ASCOT included aspects that were considered important, but according to respondents lacked coverage of health aspects as compared to the EQ-5D [2, 7, 33]. No previous study has compared how older adults interpret and respond to the ICECAP-O, ASCOT and EQ-5D.

The objective of this study was to explore the content validity and feasibility of Dutch translations of the EQ-5D-3L, ICECAP-O and ASCOT from the perspective of older adults, by identifying response issues and comparisons of coverage and comprehensibility of domains.

## Methods

### Design and sample

This is an explorative qualitative study embedded in the ACT study [34]. It focused on response issues experienced by older adults when completing Dutch translations of the EQ-5D-3L, ICECAP-O and ASCOT in order to assess the content validity and feasibility of the measures. Two cognitive interview approaches; the think-aloud technique and verbal probing, were used to identify problems originating from a mismatch between the intentions and theory behind the measures and the perspective of respondents [9, 21]. The ACT study and the amendment for this study received approval by the medical ethics committee of the VU University Medical Center (10/003).

Respondents were purposively sampled in an iterative recruitment and analysis procedure. They were selected from 3111 community-dwelling frail older adults aged 65 and above, who were previously approached for the ACT study [34], irrespective of their participation status. The purposive sample was based on a maximum variation principle, using the following characteristics: age, gender, region, PRISMA-7 score (a brief 7-item questionnaire containing risk factors for functional decline [35–37]), and, if participating in ACT measurements, educational level, presence of unmet social care needs, presence of chronic disorders and self-reported health and QoL. Potential respondents were invited for the interview by telephone, none declined to participate. Interviews took place at the home of the respondent between January and August 2012.

Recruitment was terminated after saturation was reached in the 10th interview. Saturation was determined by the number of new response issues identified in each interview [9]. After the fourth interview less than four new issues were identified per interview, and the last two consecutive interviews did not produce relevant new issues.

### Evaluated QoL measures

The measures evaluated in this study can be used in economic evaluations and describe and value QoL by several domains. They differ in the included QoL domains, the number of response options and the measurement level [5]. The format is similar: levels within each domain are described by 3–4 statements, and respondents choose the statement that best reflects their situation. We used available Dutch versions of the EQ-5D-3L [38] and ICECAP-O [39] and produced a Dutch translation of the ASCOT following forward and backward translation procedures as described by Beaton et al. [40].

#### EQ-5D-3L

The three level version of the EQ-5D is a brief 5-item instrument that measures health-related QoL by assessing aspects of physical, mental and social functioning with three response levels (no problems, some problems, extreme problems) [41, 42]. Physical functioning is encompassed in a ‘mobility’ and a ‘self-care’ item, social functioning in a ‘usual activities’ item; and mental functioning in an ‘anxiety/depression’ item.

#### ICECAP-O

The ICECAP-O was developed as an index focusing on QoL for older people rather than health [3]. Domains and terminology were derived from in-depth interviews with older people [3, 6] and were conceptually based on the capability approach [3, 6, 43, 44]. The capability approach defines wellbeing in terms of an individual’s ability to ‘do’ and ‘be’ the things that are important in life. This resulted in a measure covering five domains (one item for one domain): attachment (love and friendship), security (thinking about the future without concern), role (doing things that make you valued), enjoyment (enjoyment and plea sure), and control (independence), with four response options representing four levels of capability: none, a little, a lot and all.

#### ASCOT

The ASCOT was developed as measure of social care related-QoL. The four level self-completion version (SCT4) covers eight domains (personal cleanliness, safety, meals and nutrition, activities/occupation, control over daily life, social participation, home cleanliness and comfort and dignity) in 9 items (one item for each domain, and two items representing the Dignity domain) with four response levels (ideal state, no needs, some needs, high needs). The ASCOT aims to distinguish capabilities and functionings in the response levels, by differentiating between a no needs situation (“mustn’t grumble”) and an ideal state [7, 45].

### Procedure

Each interview started with an introduction in which the procedure and aim of the interview were explained. Before the measures were introduced, the interviewer (KvL) asked respondents to talk briefly about their current QoL and factors that influence it. Next, the interviewer asked to complete the measures, which were one by one provided to the respondent. The sequence of the measures was varied between the interviews to prevent ordering effects. The respondents were instructed to say aloud whatever they were thinking while answering the questions. The interviewer encouraged the respondents to keep ‘thinking aloud’ after moments of silence or intervened with probing questions when further clarification was desirable. This was done for instance when it was unclear how respondents arrived at their answer or when respondents verbalized thoughts that were in contrast to what was said earlier in the interview. After completing the first measurement instrument, the respondents were asked to express their opinions on the relevance and comprehensibility of the items, and about the extent to which the measure reflected their current QoL. This procedure was repeated for the other two measures. Finally, the respondents were asked to directly compare the coverage of QoL aspects and comprehensibility of the three measures. The interviewer made field notes during and after completion of the interviews. The interviews were audiotaped and transcribed verbatim.

### Analysis

We used an iterative analysis procedure; after every two interviews the transcripts were analysed. The data was initially coded by KvL. MM and AJ also read the transcripts and reviewed the applied coding. Debriefings were regularly organized to discuss findings, to reach consensus about application and definitions of codes and to refine the sample procedure.

We developed a coding scheme to categorize the response issues encountered during the interviews (as defined in Table 1). The codes were inspired by known response issues from the literature and by issues that emerged during the interviews. Concept elaboration guides and literature about the measures were used to determine deviations from intended meanings of the items. We applied constant comparative methods to analyse the data, using a similar approach as described by Knafl et al. [21]. Each item was reviewed using matrix templates in which a categorization of response issues was cross-tabulated against the list of respondents.

**Table 1:** Coding scheme

Response issue	Definition
Comprehension phase	
Understanding the words	
Odd wording	Respondent indicates that he/she finds used terms or phrases odd or unusual
Difficult wording	Respondent indicates that he/she is unfamiliar with terms or phrases or struggles with a complicated structure
Interpreting intended scope and meaning	
Difficult interpretation of item	Respondent expresses that he/she does not know or understands what is meant by the item
Wrong interpretation of item	Respondent has something else in mind when interpreting the item than intended by the developers
Narrow interpretation of item	Respondent focuses on one aspect of the construct or expresses insecurity about the focus of the item, where the intention of the developers is to cover a broad range of the construct (either due to double-barrelled items or broad multidimensional concepts)
Selecting and reporting answer phase	
Mapping	
Different answers for different aspects of item	Respondent expresses that different response options apply to different aspects of the construct (e.g. low physical control but high cognitive control) (and therefore has to choose to answer just one aspect or an average answer)
Response options partly applicable	Respondent indicates that one part of the response option fits to his/her situation but the other part not
Irrelevant response option(s)	Respondent indicates that it is impossible/unlikely that one of the response options will be chosen
Missing intermediate response option	Respondent expresses that there is a gap between two consecutive response options
Similar response options	Respondent indicates that in his/her perspective (the value of) response options is/are similar
Disagreement with order of response options	Respondent expresses or shows that the order of the response options is not in agreement with his/her perspective
Editing	
Positive responding	Respondent chooses a more positive answer than what an outsider would, based on what is known by this outsider about the respondent’s life.

Note. The categorization of codes in phases is based on Tourangeau’s model of the reponse process [17]

General opinions about the measures were analysed separately and labelled as either addressing the coverage of the measures (validity) or the comprehensibility (feasibility).

## Results

### Respondents

The variation in characteristics of the ten respondents is shown in Table 2. All respondents reported at least two health issues, varying from hearing loss to cardiovascular diseases and diabetes. Two of them indicated to have some problems with memory, attention and thinking.

**Table 2:** Characteristics of the respondents

Respondent	Age	Living situation	Region	Educational level	Frailty score (PRISMA-7)	Self-perceived health	Self-perceived QoL
Mrs. N.	90	Alone	Amsterdam	Middle	4	Fair	Fair
Mr. O.	77	With partner	West-Friesland	High	2	Fair	Unknown
Mr. Q.	100	With daughter	Amsterdam	Middle	6	Good	Fair
Mrs. S.	88	With partner	West-Friesland	Low	5	Fair	Fair
Mr. U.	75	With partner	West-Friesland	High	4	Poor	Good
Mr. W.	67	Alone	Amsterdam	Low	1	Good	Good
Mrs. X.	88	Alone	Amsterdam	Low	6	Fair	Fair
Mrs. Y.	75	With partner	West-Friesland	Middle	2	Good	Very good
Mrs. Z.	91	Alone	West-Friesland	Middle	4	Poor	Good
Mrs. A.	91	With partner	Amsterdam	Middle	6	Poor	Poor

Note. A higher PRISMA-7 score denotes a higher risk for functional decline

One respondent, Mr.Q. (pseudonyms are used), a 100-year old man, lacked the strength to complete all three measures. Mr. Q. only completed the ASCOT and his daughter helped him to elaborate on his response choices.

### Response issues

Table 3 summarizes the response issues experienced by respondents for each item of the EQ-5D-3L, ICECAP-O and ASCOT in a matrix format. The issues that occured most widely across a variety of respondents or that pose a serious threat to the validity or feasibility will be illustrated below (definitions of response issues are shown in Table 1).

**Table 3:** Matrix of issues experienced by respondents versus items of the EQ-5D, ICECAP-O and ASCOT

		EQ-5D					ICECAP-O					ASCOT								
		Mobility	Self-care	Usual activities	Pain/discomfort	Anxiety/depression	Attachment	Security	Role	Enjoyment	Control	Control over daily life	Personal cleanliness and comfort	Food and drink	Personal safety	Social participation and involvement	Occupation	Accommodation cleanliness and comfort	Dignity (filter question)	Dignity
Comprehension phase																				
Understanding the words																				
-	Odd wording												/							
-	Difficult wording																		///	/
Interpreting intended scope and meaning																				
-	Difficult interpretation of item								////		/	//					/		//////	//
-	Wrong interpretation of item				/				///											
-	Narrow interpretation of item			/////	//////	//////	///////	///////			////				//////			///////		
Selecting and reporting phase																				
Mapping																				
-	Different answers for different aspects	/	/				/				//									
-	Response option partly applicable												//			///				
-	Irrelevant response option						/								//	////				
-	Missing intermediate response options	//								/		/								
-	Similar response options									/			/		/		//			
-	Disagreement with order of response options										/			///		/				
Editing																				
-	Positive responding	//		///	//	//	////	/		//	////	///	/		/	//	/			

Note. / = issue experienced by 1 respondent

### EQ-5D-3L

#### Narrow interpretation of item

Three of the five questions from the EQ-5D-3L were more narrowly interpreted by one or more of the respondents than intended by the developers. Respondents focussed on one aspect of the double-barrelled items ‘Pain/discomfort’ and ‘Anxiety/depression’ and on one type of activities for the ‘Usual activities’ item. Where ‘Usual activities’ according to the developers encompasses social function, respondents mentioned domestic tasks and doing groceries while responding to this item. For example, Mrs. Z. (71) explained her choice [‘I have some problems with performing my usual activities’] with: “*I am not able to do many things, that is why I’ve got somebody who takes care of my household activities”.*

#### Mapping issues

Two respondents perceived a gap between response options on the ‘Mobility’ item, and considered the response options as a rough estimate, as illustrated by the comment of Mr. O. (76): “*This is either black or white, in between are many possibilities”*.

#### Positive answering

For all EQ-5D-3L items except ‘Selfcare’, it occurred that respondents picked a more positive response option than what an outsider would expect. For example, Mr. W. (67), who suffers from daily pain due to rheumatoid arthritis, chose ‘I have moderate pain or discomfort’ on the ‘Pain/Discomfort’ item because he compared his situation to that of others:

*Of course I’ve got pain, but there are thousands who have similar complaints or even worse. Pain, I don’t know any better. I get up and go to sleep with pain. It’s just as part of my life as getting coffee. I have pain but don’t feel it anymore. There are worse things in the world. When I see children in a wheelchair I always think I should not complain*.

### ICECAP-O

#### Narrow interpretation of item

Some of the ICECAP-O items were more narrowly interpreted by the respondents than the developers intended. This was most pronounced for the ‘Attachment’ and ‘Security’ items. Rather than choosing the response level that overall fitted their situation best, respondents tended to concentrate on one aspect of a domain. When answering the ‘Attachment’ question, respondents focused on friendship and did not mention love or intimacy. On the ‘Security’ item (thinking about the future without concern), respondents focused either on financial insecurities or on worries about their or their partner’s health.

#### Difficult/wrong interpretation of item

The ‘Role’ item (doing things that make you feel valued) was difficult to understand. Four respondents explicitly stated that they didn’t understand how to interpret this item. Three others provided an answer but their explanation revealed that they thought about limitations in daily functioning rather than having a purpose that is valued. Mrs. N. (89) for instance, a woman living alone who much enjoys social outings, picked the answer ‘I am able to do many of the things that make me feel valued’ and said: “*I am able to do many household tasks myself”.*

#### Mapping issues

Compared to the EQ-5D-3L, there were a few more issues with the mapping of personal situations on the available response options. For example, Mr. O. (67) indicated that he missed an intermediate response option for ‘Enjoyment’, and Mrs. X. (87) considered the last two response options of ‘Enjoyment’ as similar. Mrs. Y (74), a cheerful woman who provides informal care to her husband, ticked the most positive answer of the ‘Control’ domain [‘I am able to be completely independent’]. However, she expressed that her situation was not the most ideal in her eyes. It was an undesirable consequence of her husband’s Alzheimer’s; she rather wanted to share decisions and activities with her husband.

#### Positive answering

Positive answering occurred most often on the ‘Attachment’ and ‘Control’ items. Mrs. N. (89) talked again about household tasks while picking the most positive answer on the ‘Control’ item [‘I am able to be completely independent’]: and states “*I can do everything myself, I am completely independent, absolutely*” while she earlier explained that she receives help for chores and financial tasks from numerous people. She conveyed earlier in the interview: “*I don’t do anything in the household myself*”.

### ASCOT

#### Narrow interpretation of item

Two items were more narrowly interpreted than intended across various respondents: ‘Personal safety’ and ‘Accommodation cleanliness and comfort’. While answering the ‘Personal safety’ domain, respondents focused mainly on fear of crime, not thinking about fear of abuse, falling or physical harm. Mrs. A. (91), a woman who could not get outdoors due to the steep stairs and who during the conversation expressed fear of her dominant and aggressive husband, chose the first response option [‘I feel as safe as I want’] *“Because I always lock the door. We do this since two strange men showed up in our house. When I don’t know who’s at the door, I open the door with the door chain set in place”.* Concerning the ‘Accommodation’ item, respondents more often focused on cleanliness than on comfort of the home.

#### Difficult interpretation of item

Four items were hard to understand for several respondents. Some of them did not know which activities or aspects they ought to include or exclude in their answer on the ‘Control over daily life' and ‘Occupation’ items. Further, the ‘Dignity’ items were most poorly understood; respondents indicated they did not get what was meant by the questions and that they didn’t see a connection between having help and the way they feel and think about their selves. Mr. O. (76) decided to skip both ‘Dignity’ items:

*Which of these statements best describes how having help to do things makes you think and feel about yourself? I think that’s a difficult sentence. Please explain, because I don’t get what they mean. I completely don’t understand this question. Would you mind if I skip this one? If you wouldn’t be here I would place a big cross on the question. I don’t mind the difficult phrase, but I just don’t get it. […] I never think and feel about myself, so what do they mean?*

#### Mapping issues

Occasionally, respondents found it difficult to identify the difference between the response options. For example, Mr. W. (67) asked while responding to the Occupation item: “*What is the difference between the first two response options? This means the same to me”.*

On two other items, some respondents disagreed with the order of the response options. Three respondents, Mr. U (75), Mr. W. (67) and Mrs. Y. (74), did not perceive the first response of the ‘Food and Drink’ item [‘I get all the food and drink that I want’] as the most ideal option, as they considered that to be an unhealthy or compulsive situation.

Respondents also indicated that for two items only half of the composite response option fitted their situation. For instance, Mrs. Z. (71) chose the last response option on the Social participation item [‘I have little social contact with people and feel socially isolated’] although she indicated that she had little contact with other people, but did not feel socially isolated.

#### Positive answering

Positive answering occurred among various respondents on five items. For example, while reading the ‘Social participation’ item, Mr. Q. (100) told that he finds it very difficult that all his friends are deceased and that there is hardly anyone of his age left. Only sporadically some of his daughter’s acquaintances from church make a visit to their home. His daughter (67) confirmed that her father misses social contacts. Nonetheless, they both dismissed the two most negative response options (describing a situation with some/little social contact) because according to them these options did not fit.

### Overall comparison of the measures

Completing each interview, respondents compared the three measures based on coverage of QoL aspects (indicator of content validity) and comprehensibility of the items (indicator of feasibility).

### Coverage of QoL aspects

The respondents indicated that almost all questions of the three measures were important, and often preferred the measure which content most closely reflected their situation and daily life issues. For example, Mr. U. (74) preferred the ICECAP-O, as he spends much time reflecting on philosophical questions, and the domains covered by the ICECAP-O *“promote living with your heart”*. Mrs. Z. (71) on the other hand struggled with many physical problems and being downhearted at times, and preferred the EQ-5D-3L which health-related questions were relevant to her daily life. Mrs. Y. (74), who has no major physical concerns herself but takes care of her husband with Alzheimer, preferred the ICECAP-O and ASCOT above the EQ-5D-3L: “*These cover a somewhat broader field”.* As most of the respondents did have some health issues, the EQ-5D-3L domains were in general considered relevant, although the number of three response options gave a more “*rough indication*” than the other two measures with four response options.

While the respondents appreciated the domains of the ICECAP-O and ASCOT as they covered ‘valuable topics’, not all ICECAP-O and ASCOT domains were considered relevant. Mr. W. (67) was one of the respondents who expressed not to think about the future, therefore for him the question about ‘Security’ of the ICECAP-O was not important.

A common opinion of the respondents was that the measures did not result in a comprehensive picture of their QoL. According to the older adults, only a proper personal conversation would convey the relevant topics and details of their lives. Nonetheless, the respondents mentioned only one topic important for their QoL that was not covered by one of the measures; the concerns or delight about the wellbeing of family members, especially of (grand) children. Often the family is a source of happiness or reason for worries, and apparently the respondents did not feel that this impact on their QoL was sufficiently covered by the domains ‘Attachment’ in the ICECAP-O or ‘Social participation’ in the ASCOT.

### Comprehensibility of the items

Most consistently, the respondents indicated the EQ-5D-3L was most easy to answer, as the questions were most specific and only three response options were available, making them more distinguishable. Mr. O (76), for example, said about the ICECAP-O: “*This questionnaire is very open, all directions are possible, that is difficult for me. I think the questions should be more specific, I find this a bit too vague”*. Overall, the measure that was most relevant to the personal situation of the respondent was also the one perceived as most easy to answer.

## Discussion

This paper aimed to compare the validity and the feasibility of Dutch translations of the EQ-5D-3L, ICECAP-O and ASCOT from the perspective of older adults by identifying response issues and comparisons of coverage and comprehensibility of domains. The respondents in this study stated that their responses to the measures did not give such a comprehensive picture of their QoL as a proper personal conversation would, and specifically mentioned one domain missing in all three measures: the well-being of their family. Of the three measures, the older adults preferred, both in terms of coverage and comprehensibility of the domains, the measure that most closely reflects their daily life. Since many older adults face at least some health problems, the EQ-5D-3L is likely to be relevant to a large part of this population. Because the items in the EQ-5D-3L corresponded to the reality of their daily life, the EQ-5D-3L was also the measure most easy to complete. However, just like in other qualitative studies [2, 33] it was recognized that the ICECAP-O and ASCOT have a broader scope and include valuable topics.

We identified response issues for all items of the three measures, related to mapping of responses, interpretation of items or positive responding. These issues are likely to occur when the EQ-5D-3L, ICECAP-O or ASCOT are administered in populations of older adults, and pose potential threats to the validity and feasibility of the measures.

Mapping issues resulted in some extent to misclassification, but we expect that the effect on the validity of the responses is not large, as respondents in general chose a response alternative that most closely reflected their situation. The least amount of mapping issues were identified for the EQ-5D-3L, probably because respondents perceived the response options of the EQ-5D-3L as clearly distinguishable. The ICECAP-O and ASCOT, as well as a new five level version of the EQ-5D [46], include more response options, intended to increase the sensitivity of the measures. The results of our study suggest that more response options may be accompanied with an increase in occurrence of mapping issues.

Nonetheless, we expect that interpretation issues and positive responding pose a larger threat to the validity and feasibility of the measures. Where the EQ-5D-3L questions were interpreted most of the time as intended, the ICECAP-O and ASCOT both included questions that were poorly or wrongly understood, most apparent the ‘Role’ item of the ICECAP-O and the ‘Dignity’ items of the ASCOT. Our respondents chose to skip these items or just picked one of the answers without fully understanding what it meant. Moreover, all three measures include two or three items that were considered ambiguous, either due to double-barrelled questions or broad concepts that cover more than one domain in life. Vagueness and ambiguity can lead respondents to interpret items in variable ways [17]. This difference in interpretation would become even more troublesome when it occurs within the same person, for example when measuring QoL *changes* in economic evaluations. The impact of this response issue on the validity of responses warrants further research, especially on how it affects the measurement of changes.

Positive answering seems to appear regardless of the item or measure. This is not surprising, as older persons are notorious for giving ‘rosy’ reports of themselves and their living situation, especially to global questions [26]. These rosy reports may reflect the results of adaptation and downward comparison processes or other self-presentation and coping mechanisms [24–26, 28, 47]. Positive responding due to these mechanisms is not necessarily considered misclassification bias, for example when it reflects a real adaptation in one’s self-evaluation of QoL [47], but is a threat to validity when it occurs due to being reluctant to come across as overly negative or critical. It is important to investigate further how positive responding affects the measurement of changes.

Adaptation issues and struggles with abstract items were also found in a study that used a think-aloud approach to assess the ICECAP-A [32]. Compared to our study, a pilot study of a previous version of the ASCOT identified less response issues [7], while in another study some additional concerns about the ICECAP-A and EQ-5D-3L were expressed by research professionals [33].

Our study was the first that used a qualitative approach to compare the content validity and feasibility of the EQ-5D-3L, ICECAP-O and ASCOT in older adults. A strength of this study is that the perspective of older adults themselves was central in the assessment of the measures. Furthermore, the same methodology was used to evaluate all three measures, facilitating a comparison.

The findings of our study should be interpreted while taking the following limitations into account. First, our sampling strategy based on the maximum variation principle was aimed at identifying as many different issues as possible and limits inferences about how common the issues are in a wider population [18]. Furthermore, our sample size was quite small. The frequency of issues in Table 3 should therefore not be generalized. Although the identification of other issues in other older adults or situations cannot be excluded, we are rather confident about the comprehensiveness of the identified issues, since the number of new identified issues decreased considerably per interview. However, the sample size was too small to identify patterns between response issues and characteristics of older adults. It is likely that response issues occur more frequently among older adults with more communication difficulties, illiteracy, cognitive disorders or among those with a higher need to please others. Another limitation of the study is that the think-aloud process and the presence of the interviewer may have affected the way in which respondents answer questions, specifically the amount of attention paid to questions and the occurrence of positive responding [22, 48–50]. Finally, some of the identified issues may have occurred due to the translation of the measures into Dutch. Nonetheless, the translations were developed carefully with the intention of conceptual equivalence and entailed forward and backwards translations, as recommended.

## Conclusions

To conclude, response issues are likely to occur when the EQ-5D-3L, ICECAP-O and ASCOT are used for economic evaluations in older adults. Researchers should be aware of these issues and aim to minimize their occurrence, for example by using face-to-face interviews in studies with older adults, in which interviewers have the availability to explain items more extensively. Several response issues warrant further research, especially on the way these issues affect the measurement of changes in older adults. None of the instruments provided a comprehensive picture of the QoL of older adults in itself, but our respondents preferred the measure that most closely reflected the reality of their daily life. For now, researchers that use these measures in older adults should be aware of the response issues that may occur, when interpreting the outcomes of their study.
